# Towards an Ideology-Free, Truly Mechanistic Health Psychology

**DOI:** 10.3390/ijerph182111126

**Published:** 2021-10-22

**Authors:** Bernhard Hommel, Christian Beste

**Affiliations:** 1 Cognitive Neurophysiology, Department of Child and Adolescent Psychiatry, Faculty of Medicine, Technische Universität Dresden (TU Dresden), 01307 Dresden, Germany; Christian.Beste@uniklinikum-dresden.de; 2 Department of Child and Adolescent Psychiatry and Psychotherapy, Faculty of Medicine Carl Gustav Carus, Technische Universität Dresden (TU Dresden), 01069 Dresden, Germany; 3 Cognitive Psychology, Faculty of Psychology, Shandong Normal University, Jinan 250014, China

**Keywords:** mental health, psychiatry, ADHD, GTS

## Abstract

Efficient transfer of concepts and mechanistic insights from the cognitive to the health sciences and back requires a clear, objective description of the problem that this transfer ought to solve. Unfortunately, however, the actual descriptions are commonly penetrated with, and sometimes even motivated by, cultural norms and preferences, a problem that has colored scientific theorizing about behavioral control—the key concept for many psychological health interventions. We argue that ideologies have clouded our scientific thinking about mental health in two ways: by considering the societal utility of individuals and their behavior a key criterion for distinguishing between healthy and unhealthy people, and by dividing what actually seem to be continuous functions relating psychological and neurocognitive underpinnings to human behavior into binary, discrete categories that are then taken to define clinical phenomena. We suggest letting both traditions go and establish a health psychology that restrains from imposing societal values onto individuals, and then taking the fit between behavior and values to conceptualize unhealthiness. Instead, we promote a health psychology that reconstructs behavior that is considered to be problematic from well-understood mechanistic underpinnings of human behavior.

## 1. Introduction

The World Health Organization (WHO) defines mental health as a “state of well-being in which the individual realizes his or her own abilities, can cope with the normal stresses of life, can work productively and fruitfully, and is able to make a contribution to his or her community”. This definition is considered to be scientific in nature, but, as we argue, it reflects at least two normative, ideological premises without any scientific basis. First, the criteria of “productive and fruitful work” and especially the last part of the sentence “able to make a contribution to his or her community”, introduce the societal utility of the individual as a criterion for judging her mental health, which renders the concept of mental health an ideological rather than a medical or psychological judgment. Second, the requirement to cope with “the normal stresses of life” is just one of many examples where a normative view is introduced, contrasting what is considered to be the behavior shown by the majority of the population with the behavior of a minority that deviates from the behavioral mean to a degree that is considered problematic. This amounts to a definition of what is normal and what is not—i.e., a binary categorization of people according to some necessarily arbitrary criterion, which again is an ideological rather than a medical or psychological judgment. In the following, we criticize this ideological approach to mental health. We emphasize that this should not be taken to imply that ideologies are illegitimate, or that it is wrong to judge and treat people according to the degree to which their behavior meets normative expectations of societies. Functioning societies need norms and ideologies to regulate the peaceful and effective coexistence of their citizens, and their success in doing so will rely more on the broad acceptance of these norms and ideologies than on their fit with scientific insights. What we criticize, however, is that societal ideology has made its way into psychological modeling, into how the psychological sciences view so-called normal and abnormal behavior. We will try to demonstrate this with respect to the two mentioned premises.

## 2. Societal Utility as Criterion

The typical share of labor between the cognitive and the clinical sciences consists in the former constructing general models of human behavior, and the latter accounting for deviations from these models. Both general models and accounts of the deviations often follow a rationalistic view that has dominated psychological thinking since Plato. His tripartition of the human soul into reason, passion, and desire tries to capture what we consider the struggle between duty and pleasure: reason, the ability to follow rational thinking and proper argumentation, was considered to control the two wild horses named passion and desire. Importantly, the ability to reason was considered to be restricted to the ruling class, the citizens, while soldiers and the working-class were thought to be driven by passion and desire. This scenario was democratized to some degree by Freud, who considered people from all classes to face a continuous struggle between reason and the lower urges and instincts. The idea was that the Ego, which corresponds to Plato’s reason, negotiates between societal demands represented by the Superego and the personal passions and desires represented by the Id. More modern models of human behavior still inherit this distinction between reason and the lower urges, and the clinical sciences, especially, make ample use of it. Numerous so-called dual-route or dual-process models assume a “rational” or “intentional” route that is housed by the prefrontal cortex and tightly controlled by executive functions, which in turn are thought to represent “human will”, and another, “automatic” route that is considered to challenge the intentional route by providing action alternatives that are more fun, less effortful, more familiar, and that generate more or faster reward.

Probably the most popular example for such a dual-process model is the two-systems theory of Kahneman [1]. It contrasts “System 1”, which makes automatic, intuitive, and often emotionally based decisions that are fast and commonly rely on associations, with “System 2”, which tries to monitor and control System 1 and overrides the tendencies it might generate according to reason, logic, or probabilities. The inspiration from Plato’s horses scenario is obvious. Numerous models with this basic structure have been proposed to account for various clinical, subclinical, and otherwise unwanted behavioral phenomena, such as drug addiction [2,3], binge drinking [4], impulsive behavior [5], amoral behavior [6], stereotyping [7], and gambling [8]. The interpretation is always the same: the unwanted behavior is taken to indicate a failure of the proper balance between the two systems, that is, a lack of control by the more reasonable system and the resulting predominance of the lust-seeking one. Given that the rational/intentional system or process is considered to reflect the actual will of the agent, this lack of control is taken to imply behavior that is actually nonintentional, unwanted by the agent. This reasoning is consistent with juridical decision-making, which is also guided by the two-process idea: The more evidence for a predominance of the automatic route/process/system, the less responsible for possible damage the agent is thought to be, and the lower will be the penalty.

While this line of thinking is very popular and fits well with commonsense phenomenology, Hommel and Wiers [9] and Hommel [10] have argued that it is fundamentally flawed. First, a closer conceptual analysis revealed that almost every dual-route, dual-process, or dual-systems model uses different, and often mutually exclusive, criteria to distinguish between the two routes/processes/systems, and none of the suggested distinguishing criteria has stood systematic empirical test [11,12]. Second, the seemingly obvious logic of dual views rests on the empirically and theoretically unrealistic assumption that people (can) have only one goal at one time [10]. Consider someone engaging in unsafe sex on the occasion of a passionate one-night stand, where the means to increase safety might not be available. A classically trained health psychologist would attribute this behavior to a failure of reason: the rational behavior would be to prioritize health by avoiding the possibility of getting an infection, and possibly a very serious or even deadly one, by inhibiting one’s passions and letting this opportunity go. Accordingly, not letting it go would be considered an unintentional, irrational dominance of one’s automatic tendencies, for which the agent might therefore not be fully responsible.

As Hommel and Wiers [9] have argued, several aspects of this conclusion would be off-track. First, health is threatened by mere possibility, which can be expressed in population statistics (the probability to get AIDS from unsafe sex, for instance), but population statistics do not directly speak to this particular agent’s odds of contracting a disease from this particular encounter. Moreover, statistics can quantify risk, but do not, and cannot, determine whether this particular quantity actually matters. This problem has been obvious during the ongoing Corona pandemic, which has led to various governmental restrictions of personal freedoms. While a large majority of citizens did not question the risk of becoming infected, there have been considerable arguments regarding the amount of risk necessary to motivate particular restrictions. Take, for instance, the requirement to wear masks in public. On the one hand, there is clear scientific support for the claim that wearing masks reduces the probability of catching COVID-19 (e.g., [13], who found non-mask users to be infected at a rate of 16.4%, while mask users were infected at a rate of 7.1%). On the other hand, however, there is no objective decision rule to determine whether the population benefits obtained through the measure (the wearing of masks) justifies the restriction of personal freedom associated with implementing this measure. What if, for instance, the difference would be 2%? 1%? Moreover, people often argue that other diseases could also be prevented by similar measures, such as the flu—which, depending on the vaccination rate, can also come in waves and be very deadly for vulnerable populations. Indeed, wearing masks during the Corona pandemic has dramatically reduced these cases. While it is true that COVID-19 is more deadly, population wise, than the flu, there is no objective decision rule telling us how deadly a disease must be to justify a particular freedom-restricting measure. Accordingly, there is no scientifically justifiable parameter to decide which amount of individual risk-taking must be considered normal and which amount must not be. In other words, science can simply not objectively decide whether taking a particular risk is rational or irrational. This is why it is the role of politicians to translate scientific knowledge into societal measures.

Second, while many people consider health particularly important, not everyone does. “Live fast, love hard, die young” was a motto that country singer Faron Young sang about and that aptly described the life of musicians like Janis Joplin, Jimi Hendrix, and Jim Morrison. Health psychologists would consider lifestyles following this motto unhealthy and (therefore) irrational, which suggests that favoring a long life over an interesting life is a defining attitude in health-psychological judgments about rationality and irrationality. However, the rationality of an action can only be judged with respect to the goals and values a particular agent really has herself, so that considering a behavior irrational because it violates the interests that *other* people have can hardly be considered meaningful.

Third, and relatedly, rational choices require the weighting of the evidence in light of one’s interests. Clearly, having passionate sex is likely to be a high-priority goal for many people, and therefore having the urge to engage in some passionate sex would not necessarily be irrational or unintentional for them. It is true that weighting also requires the consideration of possible costs, and here possible diseases might play a role. However, given that there is no common currency in which the benefit of having real and immediate passionate sex can be directly compared with the relatively abstract cost of possibly attracting a disease, it is logically impossible to determine which choice actually optimizes the agent’s interest.

A possible counterargument to this reasoning from a dual-process account might be that rational considerations must always be weighted more highly than passions, so that the choice in favor of health represents the rational satisfaction of higher goals, while the choice in favor of lust represents the satisfaction of primitive urges. This is indeed consistent with Plato’s and Kahneman’s system, but this renders the choice no less ideological or more scientific. Indeed, almost all dual-process theorists attribute any behavior that can be considered societally objectionable or morally questionable to the automatic system, and everything else to the intentional one [12], as if brain evolution had equipped us with two different systems for being good and being nasty. Putting that almost religious undertone aside would leave us with an entirely different picture. If we could accept that people can have multiple goals active at the same time [14], and that some of them may favor societally acceptable actions while others may favor societally problematic actions [9], we would no longer need to consider some actions more intentional than others. Instead, we would take all actions as intentional and, accordingly, all action choices as more or less direct reflections of possible motives, irrespective of whether they are considered societally acceptable or unwanted. Accordingly, taking drugs while knowing about the accompanying risks would reflect a choice that weighs the expected drug-induced state as pleasant enough to be worth the possible risks, rather than as an indication of a breakdown of free will. An interesting implication of this view would be that even the drug addict must be considered as psychologically healthy as drug-abstinent individuals, and thus fully responsible for her drug-taking behavior. This perspective need not prevent a society from expressing its dissatisfaction with, and disapproval of, this behavior. However, the judgment would be left to the legal system rather than to clinical psychologists and psychiatrists.

With respect to the scientific explanation of human behavior, abandoning attempts to implement societal approval and disapproval of particular behaviors into the decision-making model creates the interesting opportunity of developing an integrated model that explains all kinds of behavior with the same mechanism. As elaborated elsewhere [10], both ontogenetically acquired motives and short-term goals can be considered to be represented by selection criteria that promote action alternatives that satisfy these criteria. Criteria are likely to vary in activation value, depending on the internal and external context, so that the same criterion may be dominant under some circumstances but may not contribute much under others. Given that each criterion biases action selection according to its current activation, each action is likely to satisfy various criteria that need not amount to one single homogeneous goal or intention. Accordingly, it may well be that a given individual does want to be healthy, but at the same time is very enthusiastic about the expected effects of a particular drug. The competition between the respective criteria may well result in drug-taking behavior, but the selection process as such would have the exact same characteristics as a decision against such behavior. In other words, the scientific explanation of why a particular action was eventually chosen can do without any consideration of the societal approval of that action—apart from the fact that the avoidance of expected disapproval might of course be one of the selection criteria involved in the decision-making. Note that this integrated model is compatible with the often-observed special role of habits, especially in addiction-related behaviors. However, rather than assuming that habits in some sense impose themselves on the decision-making process, the integrated model would consider the choice of habits as intentional, so to save time and effort, or to seek approval from peers. According to this logic, the question of whether a given action was driven by deliberation or by what can be considered habits or other automatic tendencies is completely immaterial for judging personal causation or legal responsibility.

## 3. Binary Classification

A second indication that ideology has made its way into our psychological models of human behavior relates to the way clinical and psychiatric cases are defined in theory and diagnosed in practice. Clinical syndromes are commonly not defined by clear-cut, objectively motivated indicators but, rather, by a range of often loosely defined, not always strongly correlated cues, often referred to as a “clinical picture” by physicians. Worse, these cues are commonly not even restricted to a particular syndrome, but shared by various others, usually for still unknown theoretical and mechanistic reasons. Take ADHD as an example. The official symptoms fall into two categories: inattentiveness and hyperactivity/impulsiveness. Some of the people diagnosed with ADHD show deviant behavior in just one category, while other people show such behavior in just the other, and yet others manifest behavior in both categories, in the absence of any coherent story explaining why this is the case. Even more problematic, the attribution of sub-symptoms of both categories follows entirely atheoretical considerations: symptoms are assumed to be present if, say, the attention span is “short”, mistakes are “careless”, talking is “excessive”, or if the diagnosed is “acting without thinking”. All of these and other criteria rely on statistics (what is the mean of the population, and how pronounced is the deviation?) and societal norms and customs. Indeed, what might be described as a short attention span or as acting without thinking might just as well be taken to express flexibility and spontaneity; what some might consider as excessive talking might be seen as entertaining and inspiring by others. Hence, almost all criteria for ADHD, and almost any other clinical syndrome of that sort, rely on purely conventional definitions of what a particular society considers appropriate and fitting.

Using such definitions from a societal point of view, to identify a particular kind of behavior that a society finds problematic and treatment-worthy would be entirely legitimate. However, using them in the hope of finding dedicated neural and functional mechanisms responsible for exactly this kind of behavior seems ill-motivated and futile. Accordingly, some efforts in psychiatry have already been devoted to replacing categorial descriptions of disorders by more dimensional criteria, or multiple dimensions to describe disorders. This has also motivated the change in nomenclature from ADHD “subtypes” (i.e., more definite traits) in DSM-IV to “presentations” in DSM-5. What remains problematic, however, is the substantial overlap of diagnostic cues related to such presentations. For example, central ADHD diagnostic criteria (e.g., distractibility, difficulty concentrating, restlessness, and avoidance of mentally strenuous activity, according to DSM-5, or inattention, hyperactivity, and impulsivity according to WHO) are also common symptoms in a number of other, often co-existing psychiatric conditions (e.g., giftedness, autism spectrum disorder). If we assume a causal connection between such cues and the functional and neural mechanisms producing them, the same mechanisms are likely to be involved in different clinical phenomena (e.g., neurotransmitter over- or under-production is considered to be a factor in many of them). However, research is organized by clinical phenomena, so that researchers working on schizophrenia submit to other journals, meet at other conferences, and are funded by other programs than researchers working on autism, for instance. This means that the overlap of cues and of their underlying mechanisms is likely to trigger the simultaneous development of similar theories in various fields, without this being noticed and fruitfully used to derive mutual theoretical and model-related constraints. In other words, relying on phenomenal categorizations of behaviors is likely to generate multiple unnecessary redundancies in our research systems.

Another problem of using atheoretically derived behavioral categories to guide basic research on human behavior consists in dividing research fields into efforts targeting “normal” behavior and efforts targeting “abnormal” behavior, often with little relation to each other. This practice follows what Lewin [15] termed Aristotelian Psychology. This kind of psychology follows the Aristotelian view that only population means deserve to be addressed by science, while variability around the mean must be considered chaos—entirely random measures that science must ignore. Our empirical approaches and our statistical methods apply this logic, as variability due to sources other than those that are experimentally manipulated is considered to be nonsystematic and theoretically irrelevant. Lewin contrasts this view with what he calls Galilean Psychology, which aims to model both population means and variability. This includes intraindividual variability—the question of why people show different behaviors at different points in time—and interindividual variability, that is, differences between people. Without also accounting for these kinds of variability, the scientific explanation of human behavior would be incomplete.

We believe that this Galilean view has enormous potential for reconceptualizing mental health. Rather than the present divide-and-conquer strategy of the clinical sciences, which first sort individuals into normal and abnormal, and then into different subcategories of abnormality, with unclear mechanistic relationships between them, the Galilean view suggests a more continuous approach, in which both “normal” and “abnormal” behavior are explained by means of the exact same mechanisms and models. To achieve this goal, it might be useful to follow what Braitenberg [16] has called a synthetic approach. Psychologists commonly follow the opposite, an analytical approach: Concepts are taken from everyday language, such as attention, memory, or action, and then, after some scientific redefinition, uncritically taken to refer to a unitary set of phenomena and processes, for which the functional and neural underpinnings are searched. The key problem with this approach is that there is no logical reason to believe that just because a word has made its way into our language, there must be a dedicated psychological mechanism that generates the phenomena the word refers to [17,18]. The synthetic approach that Braitenberg recommends turns this logic upside down. Scientific research would begin with one or more specific, well-understood mechanisms that were combined to reconstruct the phenomena one aimed to explain. The result may often not cover the entire concept but only parts of it, and this might very well be more realistic than the unfounded assumption that the mere existence of words necessarily implies a coherent set of mechanisms. Indeed, the very fact that clinical diagnostic cues often do not converge on one coherent theme may be taken to indicate that the concept is too broad, lumping together outcomes of different, perhaps even unrelated mechanisms. For instance, the considerable intraindividual fluctuations of ADHD symptoms and their great interindividual variability might reflect the heterogeneity of the levels of abstraction at which the components of the individual ADHD “fingerprint” (according to RDoC) are defined. Nevertheless, brain-based procedures in diagnosis and clinical treatment monitoring are emerging [19,20,21]. We believe that such reconstructive approaches, in which (well-understood) basic mechanisms are used to reconstruct empirical (i.e., unlabeled, uncategorized) phenomena, would be more useful and would generate less and easier-to-understand heterogeneity.

## 4. Quo Vadis?

To summarize, we consider that the current state of theorizing about mental health relies too much on ideological, normative criteria, and on arbitrary, scientifically questionable cut-off points in what seem to be rather continuous dimensions, to serve as a systematic theoretical framework for providing badly needed insight into human behavior. However, what is the alternative? We suggest that using a Galilean approach that follows a synthetic, rather than an analytic logic, might be a promising option. How does that work in practice? While we are not aware of any ongoing research line that has already implemented such an approach, our recent research on Gilles de la Tourette syndrome (GTS) has suggested to us that this approach is not only feasible, but may also lead to new, unforeseen insights that conventional analytical approaches are unlikely to offer.

GTS is a neuropsychiatric disorder characterized by multiple motor and phonic tics [22]. Due to its particularly notable motor symptoms, GTS has long been viewed and classified as a movement disorder, and treatment efficacy is indeed usually evaluated in terms of scores focusing on motor output [23]. However, several lines of research reviewed elsewhere [24,25,26,27] have reported numerous non-motoric peculiarities of GTS patients, such as hypersensitivity to external stimuli [27] and general perceptual processing [28], abnormal sensorimotor interaction [29,30], and a dependence of symptoms on attention [31,32,33]. Moreover, the degree to which motor symptoms can be controlled [34] has been reported to form the basis of cognitive–behavioral interventions, and an increased tendency to create habits has been observed [35,36,37]. These and other various lines of research suggest that GTS is not an unequivocal movement disorder, but a disorder in which perception–action integration and cognitive control processes also play a role [24,38,39,40,41,42,43,44].

Rather than attempting to identify the one functional or neural factor that might be responsible for all these observations, Beste and his colleagues consulted general models of perception and action, in an attempt to see whether the obtained peculiarities could be reconstructed from the general principles spelled out in these models [24,39]. A model that emerged as particularly useful for this purpose, due to its clear mechanistic description of the representations and processes involved in perception, action, and sensorimotor integration, was the theory of event coding (TEC [45]). TEC assumes that the binding between perception and motor responses (actions) occurs in so-called event files [46], and it allows for a theoretically stringent analysis of perception–action integration and the theory-guided experimental test of hypotheses regarding perception, action, and perception–action integration. On the one hand, TEC failed to provide one particular mechanism that could account for all, or at least all central peculiarities that characterize GTS. On the other hand, TEC did allow for the systematic experimental investigation of the mechanisms underlying each peculiarity separately, without the presupposition that all of them needed to converge on one single network, system, or function. For instance, associations between sensory and motor processes were found to predict tic frequency [47,48], and the role of habit formation in GTS could be accounted for [49]. Along the same lines, it was possible to explain why cognitive behavioral interventions are likely to be effective [43]—which would be odd if GTS really reflected a movement disorder. Hence, the coherence of our insights into GTS was not provided by the clinical definition, but by the theory that specified the roles of each mechanism involved in GTS in human perception and action control. This allowed us to translate theory-driven experiments into a clinical setting, and to account for each component that clinical phenomenology refers to, without assuming any kind of homogeneity in the phenomenology or the mechanistic causes generating it.

Let us consider the various neurobiological underpinnings in more detail. Various lines of evidence concerning GTS pathophysiology suggest that inhibitory–excitatory imbalances between the partially segregated cortico-striato-thalamo-cortical loops [50] are central for the understanding of GTS [50,51]. Reported structural abnormalities in GTS encompass cortical and subcortical structures associated with motor, behavioral, and emotional control, including the prefrontal and cingulate cortex, the basal ganglia, the sensorimotor cortex, and the corpus callosum [52,53,54,55,56,57,58]. At the network level, the involvement of circuits including prefrontal areas, the supplementary motor area (SMA), the basal ganglia and primary motor cortex (M1), and frontoparietal networks has been proposed [59,60]. These findings documenting reduced large range intracortical connections have been interpreted as a sign of a less “mature” long-range cortical network architecture in GTS [61]. Abnormalities in cortico-striato-thalamo-cortical circuits in GTS are related to changes in the dopaminergic system, which is why dopaminergic treatments [62] are still a mainstay for the therapy [41,63]. Tics are probably related to a hyperdopaminergic state [41]. The GABAergic and the norepinephrine (NE) systems have also been shown to be altered in GTS [64,65,66,67,68]. Clonidine and Guanfacine, α2 agonists, can improve tics [69,70,71,72], but there is also an ongoing discussion about the role of immunological factors in GTS [73,74]. However, several studies suggest that event file coding is modulated at a striatal level. There are strong links between the updating of mental sets and different dopamine-modulating genotypes that significantly influence the amount of striatal dopamine [75] or striatal dopamine receptor density [76]. More specifically, it has been shown that striatal dopamine levels modulate the flexible management of stimulus–response associations [77,78]. The cognitive view is thus commensurable with neurobiological views on the pathophysiology of GTS.

Future studies are needed to unify the cognitive and neurobiological viewpoints into a coherent framework. We emphasize that the example of GTS was not the result of a conscious implementation of an approach that we suggest here but, rather, the result of an extended struggle aimed at the better integration of basic-science cognitive modeling on the one hand, and a deeper understanding of clinical observations on the other. Nevertheless, we believe that it does show that such an integration is both possible and fruitful, and that a less conservative, less ideologically biased approach is of value in addressing clinical phenomena.
